# Relationship of Indicators of Cardiovascular Health With Change in Cognition in Older Blacks

**DOI:** 10.1093/geroni/igaa057.2827

**Published:** 2020-12-16

**Authors:** Melissa Lamar, Debra Fleischman, Sue Leurgans, Konstantinos Arfanakis, Lisa Barnes

**Affiliations:** 1 Rush University Medical Center, Chicago, Illinois, United States; 2 Rush Alzheimer’s Disease Center, Chicago, Illinois, United States

## Abstract

Older Blacks perform more poorly on cognitive testing than older Whites. Increased prevalence of cardiovascular disease risk factors (CVD-RFs) among Blacks compared to Whites contribute to these disparities. We investigated whether white matter hyperintensities (WMHs) in late-life, a consequence of mid-life CVD-RFs, modify the association between late-life CVD-RFs and cognition in 167 Blacks (age~75yrs; non-demented at baseline). Participants were evaluated for blood pressure (BP) markers of cardiovascular health (systolic/diastolic BP, pulse pressure, mean arterial pressure (MAP) hypertension), WMH volumes adjusted for intracranial volume, and cognition (global and domain-specific at baseline and annually, ~8yrs). Adjusted models revealed differential associations between BP markers and baseline cognition; diastolic BP and MAP predicted faster decline in episodic memory. Hypertension was not significant in any model; however, when adding WMH, the hypertension*WMH interaction was significant for baseline cognition. Cognition among older Blacks is a complex function of BP markers of cardiovascular health and WMH.

